# Pregabalin Versus Duloxetine on Postoperative Analgesic Requirement Following Lower Extremity Trauma Surgeries: A randomised, Clinical Trial

**DOI:** 10.5152/TJAR.2022.21098

**Published:** 2022-10-01

**Authors:** Alisha Chachra, Vanita Ahuja, Deepak Thapa, Satinder Gombar, Ravi Gupta, Sandeep Gupta

**Affiliations:** 1 Department of Anaesthesia and Intensive Care, Government Medical College and Hospital, Chandigarh, India; 2 Department of Orthopaedics, Government Medical College and Hospital, Chandigarh, India

**Keywords:** Duloxetine, lower limb trauma, postoperative analgesia, pregabalin, Ramsay sedation score

## Abstract

**Objective:**

The use of pregabalin versus duloxetine in postoperative lower limb traumatic pain has not been compared. The aim of this study was to evaluate the response rate of rescue analgesic requirement with perioperative pregabalin versus duloxetine in lower limb trauma surgeries.

**Methods:**

In this randomised, clinical trial, 60 patients of American Society of Anesthesiologists physical status I-II undergoing lower limb trauma surgery were randomised to receive oral pregabalin 150 mg day^-1^ or duloxetine 60 mg day^-1^, 2 hours prior to surgery and then once daily for next 2 days postoperatively. The surgery was performed under standardised spinal anaesthesia technique. The investigator was blinded to the study drug, oral paracetamol 1 g every 6 hours and intravenous diclofenac 75 mg was a rescue analgesic. The primary outcome of the study was response rate in terms of rescue analgesia requirement. Secondary outcomes included total rescue analgesia, visual analogue scale at rest and on movement, haemodynamics, anxiety depression score, and patient satisfaction score and adverse effects.

**Results:**

In group pregabalin, 60% of patients required the first dose of rescue analgesia versus 50% in group duloxetine for 72 hours postoperatively. In group pregabalin, 6.6% of patients required the second dose of rescue analgesia after a mean duration of 24 hours, and 10% of patients in group duloxetine required the second dose after a mean duration of 40 hours. The visual analogue scale scores, time to first rescue, and cumulative rescue analgesic were comparable in both the groups.

**Conclusion:**

Equivalent rate-responsive rescue analgesia was required in patients receiving pregabalin or duloxetine following lower limb trauma surgery.

**Main Points**

- The study reported reduced patient health questionnaire and generalised anxiety disorder in patients receiving either pregabalin or duloxetine. Similar patient satisfaction score and length of hospital stay were observed in both the groups.
- Published literature reports only one study comparing the use of pregabalin versus duloxetine for pain relief in lumbar disc herniation surgery. Since this was the first study comparing pregabalin and duloxetine in lower limb trauma patients, studies with larger sample size and longer duration may be planned in the future for evaluating postoperative analgesia.

## Introduction

Lower limb trauma comprises a significant portion of trauma surgeries causing moderate to severe postoperative pain. Trauma involves damage to peripheral nerves by compression or crush.^1^ The nerve damage sets off a cascade of inflammatory changes with chemical, structural, and functional changes in peripheral and central nervous systems. Uncontrolled pain can lead to peripheral and later central sensitisation.^2^ The multimodal analgesia includes non-steroidal anti-inflammatory drugs (NSAIDs), opioids, gabapentinoids, selective serotonin reuptake inhibitors (SSRIs), and regional nerve blocks. Oral and intravenous analgesics are administered routinely in postoperative period for multimodal pain relief.^3,4^

Pregabalin has been used for peripheral neuropathic pain, diabetic peripheral neuropathy, fibromyalgia, post-herpetic neuralgia, and acute postoperative pain.^5^ The analgesic efficacy of newer classes of drugs like SSRIs, namely, duloxetine, venlafaxine, and milnacipran, has been utilized in chronic lower back pain, somatoform disorders, diabetic neuropathy, fibromyalgia, and non-cardiac chest pain.^6^ The literature is scant in the comparison of the efficacy of pregabalin and duloxetine in lower limb trauma patients.

Hence, the present study was carried out to compare the postoperative analgesic efficacy of pregabalin and duloxetine in lower limb trauma patients. The primary outcome of the study was to compare the proportion of patients requiring rescue analgesia in 2 different groups receiving either pregabalin or duloxetine. The secondary outcomes of the study were total cumulative rescue analgesia consumed, visual analogue scale (VAS) at rest, VAS on movement, patient demographics, haemodynamics, patient health questionnaire (PHQ) and generalised anxiety disorder (GAD) score, nausea and vomiting, Ramsay sedation score, patient satisfaction score, and patients were followed up to 3 months for the development of chronic pain.

To our knowledge, this is the first study to compare the rate-responsive rescue analgesic consumption of diclofenac in patients receiving oral pregabalin or duloxetine up to 72 hours postoperatively in lower limb trauma.

## Methods

This randomised, clinical trial was conducted in the Department of Anaesthesia and Intensive Care, in collaboration with the Department of Orthopaedics, in a Tertiary care hospital. Before data collection, approval of the hospital ethics committee (EC/2018/156, dated December 26, 2018) and registration of the Clinical Trial Registry (CTRI/2019/02/017660 dated 14-02-2019) were taken. The design and conduct of our trial adhered to the Consolidated Standards of Reporting Trials (CONSORT) statement, Declaration of Helsinki, and Good Clinical Practice Guidelines.

**Figure 1.**
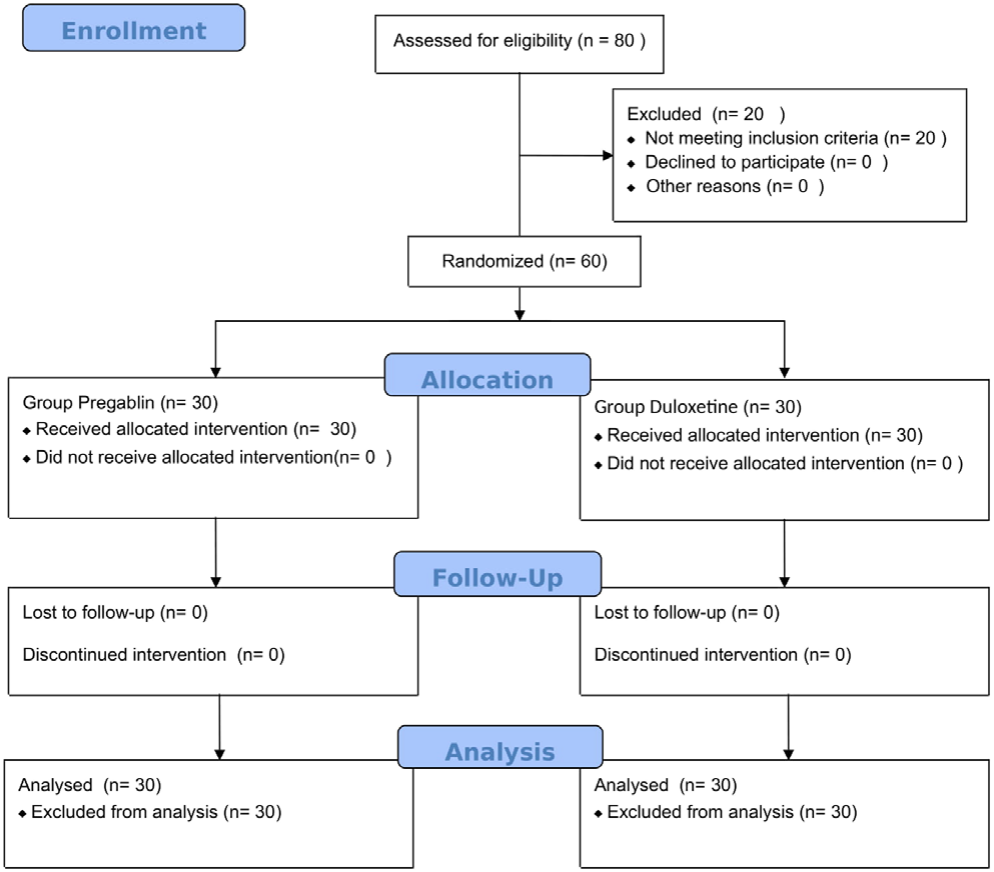
Consort diagram.

All American Society of Anesthesiologists (ASA) physical status I-II, trauma patients of 18-60 years of age, belonging to either gender, scheduled to undergo lower limb trauma surgery for recent fracture (within 1 month) of unilateral femur or tibia under subarachnoid block were included. The exclusion criteria were patients with head injury and/or polytrauma including bilateral fractures, coagulopathy, PHQ-9 and GAD-7 score >4, patients on medications for chronic pain, pregnant/lactating women, patients unable to understand VAS, patients with substance abuse, and patients with contraindications to pregabalin, duloxetine, and diclofenac. Randomisation of patients was done using a computer-generated random number table. The consort diagram is presented in Figure 1.

### Preparation of the Patient

All the patients were assessed a day prior to surgery to evaluate fitness for surgery under spinal anaesthesia. The required blood investigations, that is, haemogram, coagulogram, serum electrolytes, renal function tests, electrocardiogram, and chest X-ray were done in all the patients. Randomisation of patients was done using computer-generated random number table generated by an anaesthesiologist (AC) not involved in the study. Patients were enrolled in the study by anaesthesiologist after fulfilling inclusion and exclusion criteria into 1 of the 2 groups:

Group pregabalin (control group): 30 patients were given 150 mg pregabalin 2 hours prior to surgery and once a day for 2 days after the surgery.

Group duloxetine (intervention group): 30 patients were given 60 mg duloxetine 2 hours prior to surgery and once a day for 2 days after the surgery.

The group allocation envelope with the study drug was handed over to the staff nurse present in the ward. The nurse administered the drug as per written instructions provided in the envelope to the patient. Concealment was done by providing patients with sequentially numbered opaque-coloured sealed envelopes containing the study drug as per group allocation. The assessor (AC) was blinded to the group allocation.

### Procedure on the Day of Surgery and Monitoring

In the operating room, a multichannel anaesthesia monitor (AS5, Datex Ohmeda, Finland) was attached to the patient and baseline parameters, that is, heart rate (HR), mean arterial pressure (MAP), electrocardiogram (ECG), and arterial oxygen saturation (SpO_2_), were recorded. An intravenous (IV) access in the dorsum of the hand was established, and the patient was preloaded with 500 mL of normal saline. Under all aseptic precautions, subarachnoid block was performed with 3 mL of 0.5% bupivacaine heavy and 25 µg of fentanyl, and a final volume of 3.5 mL was injected at the level of interspace L_2_-L_3_ or L_3_-L_4_. Towards the end of surgery, IV paracetamol of 1 g was administered and then patient was shifted to post anaesthesia care unit (PACU). Subsequently, in the ward, all patients received oral paracetamol of 1 g every 6 hours up to 72 hours postoperatively, and the study drug was given once a day for the next 2 days. If the patient reported a VAS score on the movement of ≥ 4, then IV diclofenac of 75 mg in 100 mL normal saline was administered as a rescue analgesic. Postoperative pain was assessed on VAS which is an 11-point scale with 0 = no pain to 10 = worst imaginable pain.^7^ Preoperative anxiety and depression score was assessed with PHQ-9 score of 9 questions with a total ranging from 0 to 27 and GAD-7 score of 7 questions with a total ranging from 0 to 21.^8^ Ramsay Sedation Scale^9^ was used to assess sedation on a 6-point scale from 1 to 6. Nausea and vomiting scale^10^ was used to assess nausea and vomiting on a 4-point scale from 0 to 3. Patient satisfaction score^11^ was used postoperatively at 72 hours. The patients were asked to rate the level of satisfaction using a 5-point scale ranging from 1 to 5. Verbal numeric rating scale (VNRS)^12^ was used for assessing the pain of the patient telephonically at 3 months postoperatively.

### Clinical Outcome

Primary outcome of the study was response rate in terms of analgesic requirement which was recorded from the immediate postoperative period till 72 hours postoperatively. The secondary outcomes included total cumulative rescue analgesia consumed during the entire 72 hours postoperatively, VAS at rest and on movement preoperatively, and from immediate postoperative period till 72 hours postoperatively in the ward,^7^ patient demographics,^13^ haemodynamics including heart rate, blood pressure, mean arterial pressure preoperatively, and from immediate postoperative period till 72 hours postoperatively in the ward. Anxiety and depression score using PHQ-9 and GAD-7 was assessed during preoperative visit and at 72 hours postoperatively in ward.^8^ Nausea and vomiting were assessed on a 4-point scale from immediate postoperative period till 72 hours postoperatively in the ward.^10^ Ramsay sedation score was assessed preoperatively and from immediate postoperative period till 72 hours postoperatively in the ward.^9^ Patient satisfaction score was noted at 72 hours postoperatively in ward.^11^ Lengths of hospital stay were assessed at the time of discharge. Verbal numeric rating scale at rest, VNRS on movement, and patient satisfaction score were assessed at 3 months follow-up. All these observations of all the patients were recorded. Decoding was done at the end of the study and analysed statistically using appropriate statistical tests.

### Statistical Analysis

Optimum sample size was calculated on the basis of pilot cases. The reported proportions of patients requiring rescue analgesia were 40% patients in pregabalin group and 80% patients in duloxetine group. Assuming 5% as the level of significance with a power of 80%, the optimum sample size for comparison of these two study groups, the number of subjects per group came out to be 23. For any possible dropouts, 30 patients in each group were included, and thus total sample size was 60 patients. Continuous data were represented as mean ± standard deviation or median and interquartile range, as appropriate. Normality of quantitative data was checked by measures of Kolmogorov–Smirnov tests of normality. For skewed data or scores, comparisons for 2 groups were made by Mann–Whitney test. For normally distributed data, Student’s *t*-test was applied to compare the 2 groups. Categorical variables were reported as counts and percentages. Group comparisons were made with the chi-square test or fisher’s exact test. For time-related variables of skewed data, Wilcoxon signed-rank test was applied, and for normally distributed data, analysis of variance followed by post hoc multiple comparisons test (Bonferroni correction) was carried out. Survival curves were constructed by Kaplan–Meier curve and were compared by log-rank method. *P* <.05 was considered significant. Analysis was conducted using Statistical Package for the Social Sciences Statistics version 22.0 (IBM Corp.; Armonk, NY, USA).

## Results

A total of 80 patients were screened. Of these, 60 fulfilled the inclusion criteria and were operated on for lower limb trauma. Patient characteristics were similar in both the groups as presented in Table 1. All the patients were operated on for fracture shaft of femur or tibia within 1-2 days of trauma.

**Figure 2.**
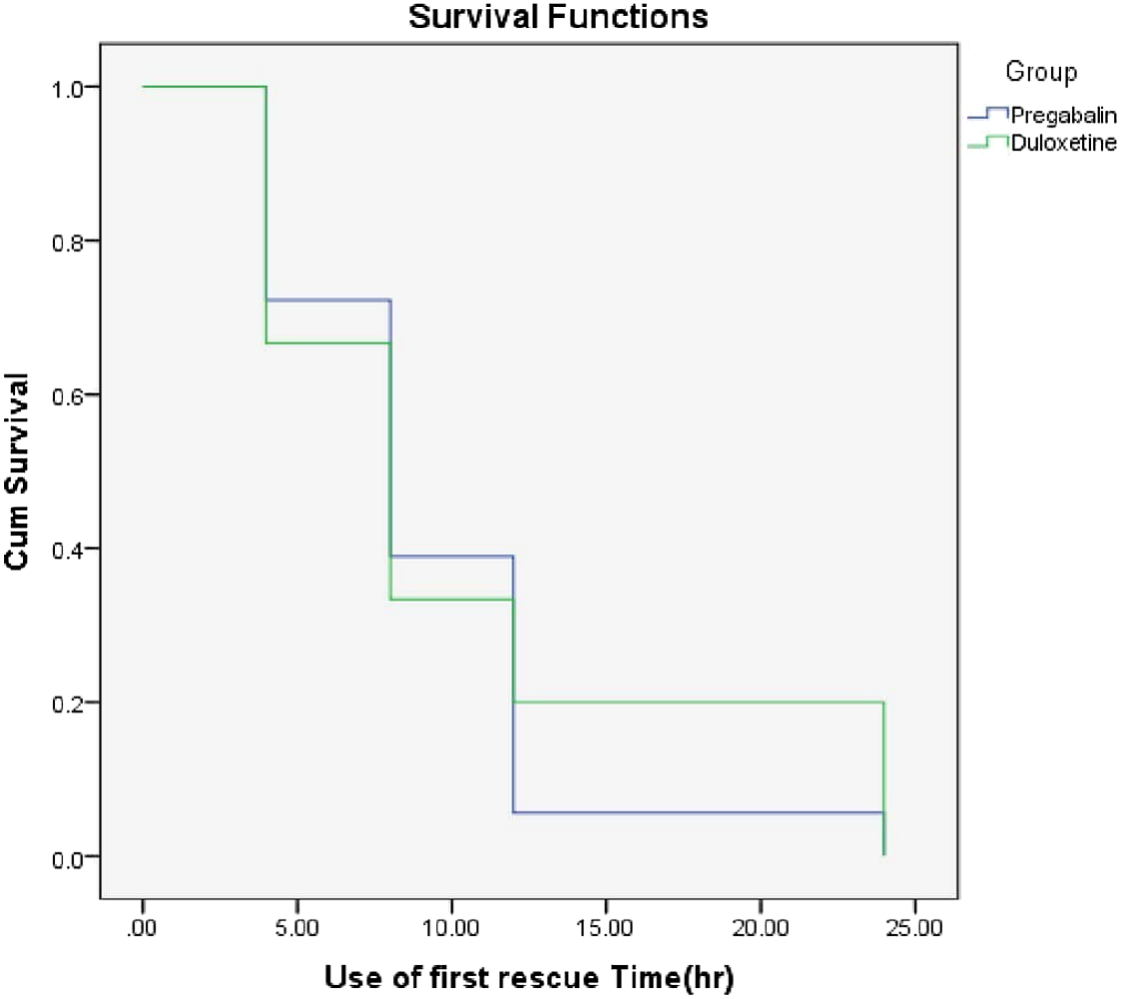
Kaplan Meier curve depicting the time to first rescue analgesia in group pregabalin versus group duloxetine.

In group pregabalin, 60% of patients required the first dose of rescue analgesia versus 50% in group duloxetine for 72 hours postoperatively. While in group pregabalin, 6.6% of patients required the second dose of rescue analgesia after a mean duration of 24 hours and 10% of patients in group duloxetine required the second dose after a mean duration of 40 hours. The median time to first rescue analgesia in both groups was 8 hours as shown in Figure 2, Table 2.

**Figure 3.**
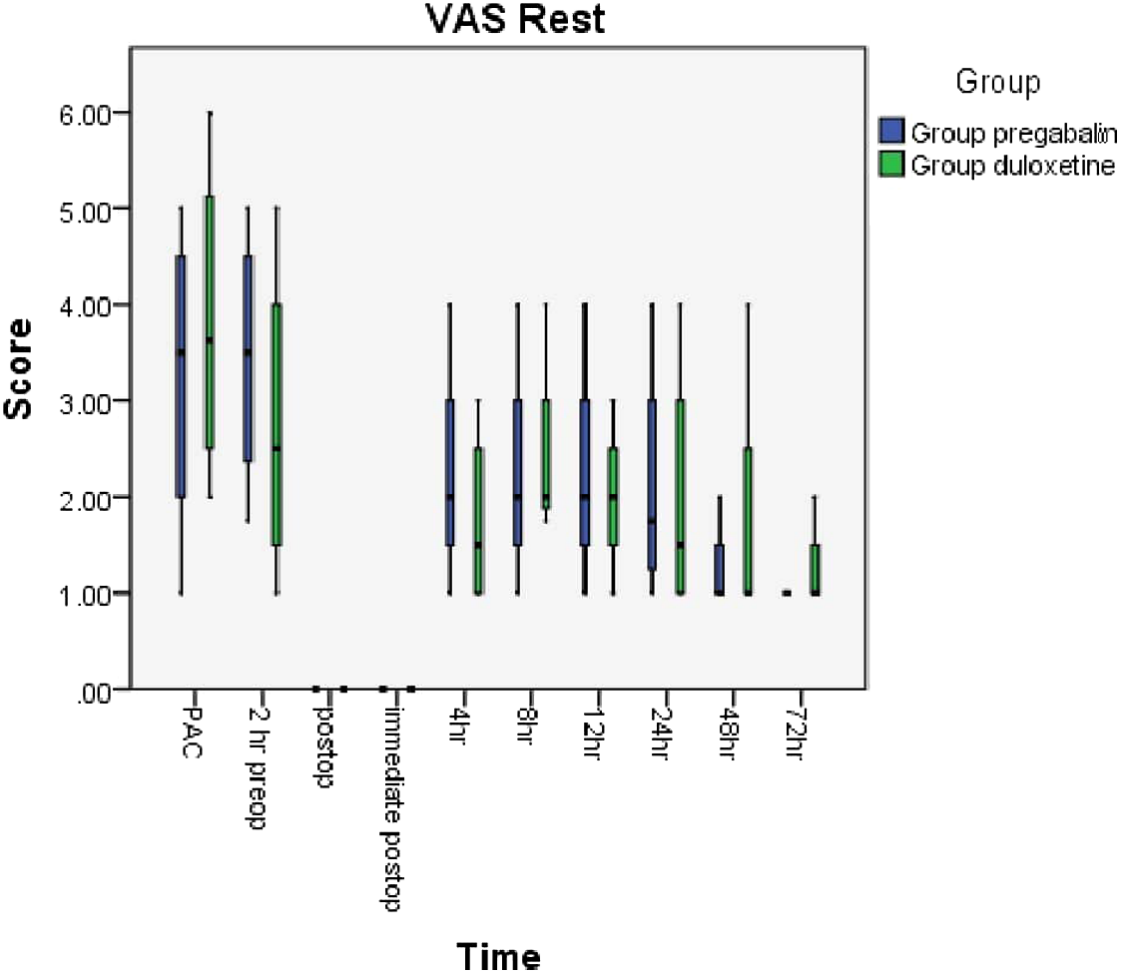
Box whisker plot showing comparison of visual analogue scale (VAS) at rest in group pregabalin versus group duloxetine.

**Figure 4.**
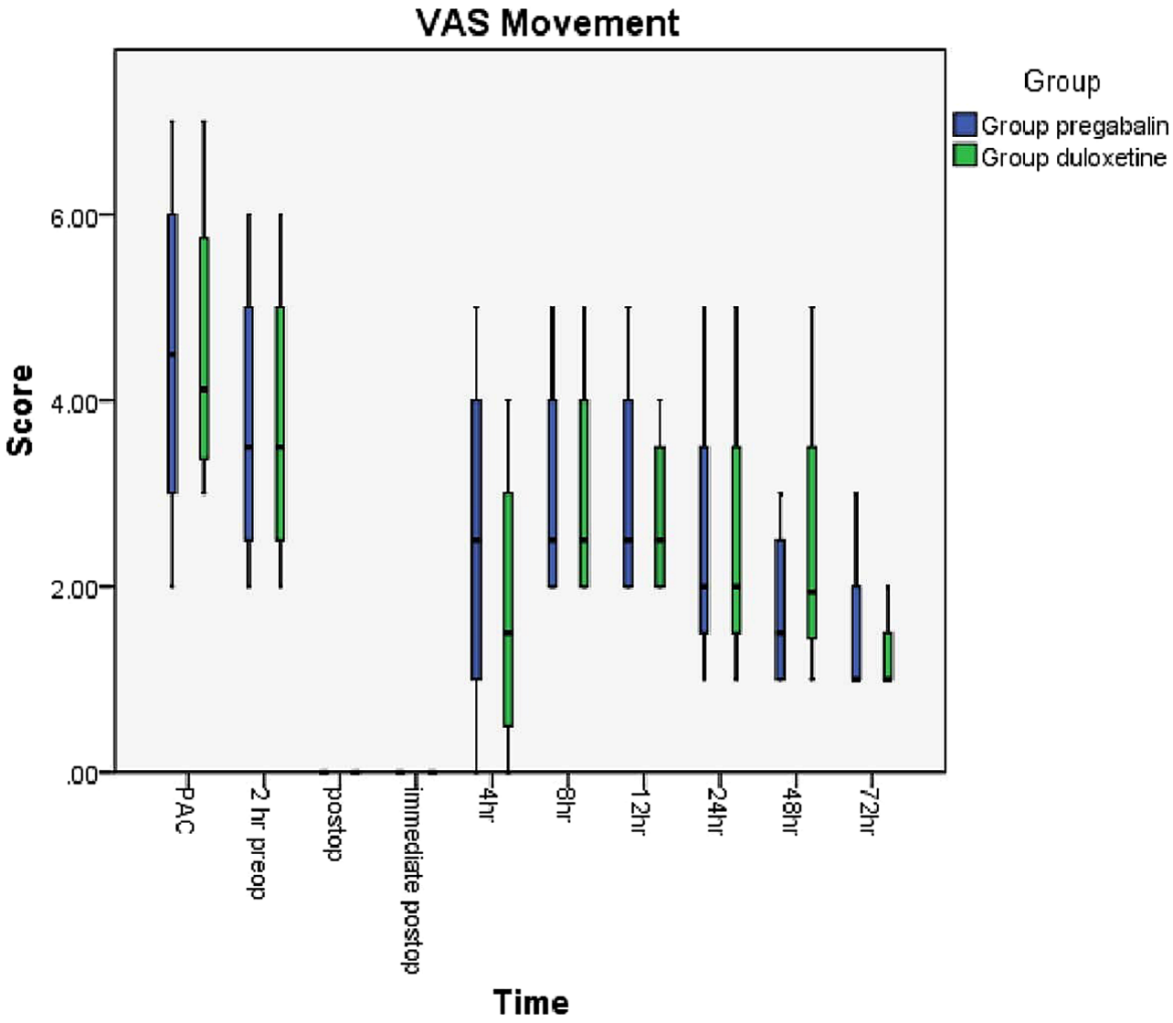
Box whisker plot showing comparison of visual analogue scale (VAS) on movement in group pregabalin versus group duloxetine.

The median VAS at rest and on movement was comparable in both the groups as depicted in Figures 3 and 4. The median postoperative PHQ score was 0.00 [0.07-0.39 (0-1)] in group pregabalin and 0.00 [0.13-0.54 (0-2)] in group duloxetine. The values were comparable in both the groups postoperatively (*P* = .52). The postoperative GAD score in group pregabalin was 0.00 [0.03-0.31 (0-1)] and in group duloxetine it was 0.00 [0.03-0.51 (0-2)] (*P* = .86).

The haemodynamics were comparable and within normal physiological limits at all time intervals between both the groups. Ramsay sedation score was comparable in both the groups except at 4 hours which was 2.00 [2.00-4.00 (1-4)] in group pregabalin and 4.00 [2.00-4.00 (2-4)] in group duloxetine (*P* = .03). At 48 hours, Ramsay sedation score was 2.00 [2.00-4.00 (2-4)] in group pregabalin and 2.00 [2.00-4.00 (2-4)] in group duloxetine (*P* = .005). One patient in group pregabalin and 2 patients in group duloxetine reported nausea and vomiting during the entire study period. There were no major adverse effects reported. Three patients in group duloxetine complained of drowsiness. One patient each of group pregabalin and group duloxetine reported slight dizziness.

The median duration of hospital stay was comparable in both the groups. All patients were followed telephonically up to 3 months postoperatively with no loss to follow-up. The patient satisfaction score was comparable in both the groups at 72 hours and at 3 months follow-up. The VNRS at 3 months was comparable in both the groups.

## Discussion

In this study, we found a comparable rate-responsive rescue analgesic consumption of diclofenac in patients receiving oral pregabalin or duloxetine up to 72 hours postoperatively in lower limb trauma surgeries.

Trauma has a component of tissue and nerve damage. Pregabalin acts predominantly through a centrally mediated action via the cerebral cortex and periaqueductal gray, while duloxetine acts via noradrenergic and serotonergic neurons in the descending spinal pathway on the dorsal horn.^14,15^ Published literature reports only one study comparing the use of pregabalin versus duloxetine for pain relief in lumbar disc herniation surgery.^16^ Altiparmak et al^16^ reported that the majority of the patients required 3 rescue analgesics, that is, 86.7% of patients in pregabalin group and 87.1% of patients in duloxetine group during a period of 24 hours postoperatively. But in the present study, only 60% of patients in group pregabalin and 50% of patients in group duloxetine required the first dose of rescue analgesia and only 6.6% of patients in group pregabalin and 10% of patients in group duloxetine required the second dose of rescue analgesia in 72 hours. None of the patients required third rescue analgesic in the present study. Altiparmak et al^16^ used a higher dose of pregabalin of 225 mg and duloxetine of 120 mg in contrast to 150 mg of pregabalin and 60 mg of duloxetine in the present study. Altiparmak et al^16^ included a control group; however, in the present study, no control group was used since both pregabalin and duloxetine have been used in postoperative period and a head to head comparison was the lacunae in the existing literature. Altiparmak et al^16^ reported time to first rescue analgesia, 221.00 ± 71.12 minutes in pregabalin and 211.45 ± 69.75 minutes in duloxetine group, while in the present study, the median time to first rescue was 8.00 [4.00-12.00 (4-24)] hours (~480 minutes) in both group pregabalin and group duloxetine. However, the authors have not commented on the cumulative mean dose of diclofenac rescue analgesia, but on extrapolation as majority of patients demanded third dose of rescue analgesia in group pregabalin and in Group Duloxetine, the requirement of diclofenac was much higher^16^ than the present study. Altiparmak et al^16^ concluded that VAS reduction was comparable with pregabalin and duloxetine, which is in support of the present study, but the requirement for rescue analgesic was much higher in spite of higher dosage of pregabalin and duloxetine administered by Altiparmak et al.^16^ The reasons could be that firstly, the type of surgery and secondly, the type of anaesthesia. In the present study, lower limb trauma surgery was operated on under SA versus lumbar spine surgery under GA in Altiparmak et al.^16^ Altiparmak et al^16^ compared postoperative cognitive function using MoCA and Ramsay sedation score was used to assess postoperative sedation in the present study. In the present study, PHQ and GAD scales were used to evaluate the mental state of a participating patient. Sometimes a previous disturbing event or unsuitable place environment may influence the psychological and mental state of a patient even later in life. This can be reflected as continuity anxiety. To gauge anxiety and depression, a single instrument may not be sufficient, and thus, studies using several scales in combination are recommended.^17^ The 2 scales PHQ and GAD were used in the present study to determine the psychological anxiety and depression status of patients in the study. This was important as pain being a multidimensional entity; we wanted that all patients had similar level of baseline PHQ and GAD. This was done to ensure that there was no influence of mental state on the outcome of pain relief following pregabalin versus duloxetine.

The published literature reports the use of pregabalin or duloxetine with placebo.^18-21^ The metanalysis of use of pregabalin 100-300 mg day-1 in patients undergoing primary total knee arthroplasty (TKA) and primary total hip arthroplasty (THA) have reported reduced VAS score in patients between 24 and 48 hours.^18^ In another meta-analysis of 10 studies, preoperative oral pregabalin of 75-600 mg day-1 reduced postoperative pain, morphine consumption, and PONV in patients undergoing hysterectomy.^19^ Regarding duloxetine, perioperative duloxetine of 60 mg day-1 given for 2 days reduced postoperative morphine consumption and postoperative pain in 50 patients undergoing TKA under general or neuraxial anaesthesia in comparison to a placebo group.^20^ A better analgesic efficacy of oral duloxetine of 60 mg with IV dexamethasone of 0.1 mg day-1 was reported as compared to duloxetine of 60 mg alone on the basis of postoperative analgesia requirement and lesser postoperative side effects.^21^ Reduced postoperative morphine requirement was reported with the use of oral duloxetine of 30-60 mg for 1 week.^22^ Castro Alves et al^23^ reported better emotional and physical comfort with duloxetine in a randomised, placebo-controlled, double-blinded trial in patients undergoing abdominal hysterectomy. We used a dose of oral pregabalin of 150 mg and duloxetine of 60 mg as supported by published studies.^24,25^ However, studies evaluating for 72 hours postoperatively in lower limb trauma patients are not reported.

The choice of oral pregabalin of 150 mg or duloxetine of 60 mg shall depend not only on the pharmacokinetic and pharmacodynamic properties but also on dosage per day, comorbidities of the patient, and adverse effects and interaction with other drugs influencing serotonin syndrome. Both drugs act centrally through different mechanisms and have unique pharmacodynamics. Pregabalin follows linear pharmacokinetics. The peak effect of pregabalin is achieved in 1-2 hours, while for duloxetine, it is 6 hours. The plasma protein binding for pregabalin is assumed to be 0, and duloxetine is 96% bound to plasma proteins. Volume of distribution for pregabalin is 0.5 L day-1 and 1.6 L day-1 for duloxetine. The oral bioavailability of pregabalin is >90%, but the half-life is 5.5-6.7 hours. On the contrary, duloxetine is 50% bioavailable due to hepatic metabolism but has longer half-life of 10-12 hours.^14,15^ Although it has been shown that even a single dose of these drugs reduces the need for postoperative analgesic, it should be used longer for analgesic efficacy, especially in neuropathic pain. Hence, in the present study, there were similar results in both groups due to the use of equipotent dosage and patients had normal renal and liver function test. Moreover, pregabalin or duloxetine was administered for 72 hours, a longer duration may influence the results.

The study period for the use of pregabalin and duloxetine in lower limb trauma patients was limited to 72 hours postoperatively. Studies with larger sample size and longer duration may be planned in the future for evaluating postoperative analgesia.

## Conclusion

The study established a similar rate-responsive rescue analgesic consumption in patients receiving perioperative pregabalin versus duloxetine up to 72 hours postoperatively following lower limb trauma surgery under spinal anaesthesia.

## Figures and Tables

**Table 1.** Baseline Characteristics of 60 Patients Receiving Pregabalin Versus Duloxetine in Lower Limb Trauma Surgeries

**Patient Characteristics**	Group Pregabalin (n = 30)	Group Duloxetine (n = 30)	*P*
Age (years)	28.20 ± 10.07 [24.44-31.96 (18-50)]	31.53 ±7.76 [28.67-34.40 (19-48)]	.15
Height (cm)	168.85 ± 8.05 [165.84-171.86 (152.4-183)]	171.75 ± 6.41 [169.36-174.15 (155.44-180.34)]	.13
Weight (kg)	59.53 ± 7.55 [56.71-62.35 (45-80)]	63.27 ± 8.18 [60.21-66.32 (45-80)]	.07
BMI (kg m^-2^)	21.50 ± 2.76 [20.46-22.52 (17.0-27.6)]	22.43 ± 3.21 [21.24-23.63 (17.3-31.1)]	.23
Socioeconomic status-2	8 (26.7)	3 (10.0)	.24
Socioeconomic status-3	8 (26.7)	11 (36.7)	
Socioeconomic status-4	14 (46.7)	16 (53.3)
ASA-I/II	24 (80.0)/6 (20.0)	23 (76.7)/7 (23.3)	.75
Males/ Females	25 (83.3)/5 (16.7)	29 (96.7)/1 (3.3)	.08
Preoperative PHQ	2.00 [1.18-2.54 (1-4)]	2.00 [2.05-2.62 (1 -4)]	.40
Postoperative PHQ	0.00 [0.07-0.39 (0 -1)]	0.00 [0.13-0.54 (0 -2)]	.52
Preoperative GAD	1.00 [1.03-1.63 (0-3)]	2.00 [1.24-2.03 (0-4)]	.29
Postoperative GAD	0.00 [0.03-0.31 (0-1)]	0.00 [0.03-0.51 (0-2)]	.86
Fracture shaft of femur	8 (26.7)	8 (26.7)	.61
Fracture shaft of tibia	22 (73.3)	22 (73.3)
Length of hospital stay (days)	3.00 [3.00-3.25 (3-20)]	3.00 [3.00-3.25 (3-5)]	.94

Values are represented as mean ± SD or number (%) or median [IQR (range)]. Student’s *t*-test was used for Age, weight, height, BMI gender, socioeconomic status, ASA grade. Chi-square/Fisher’s exact test for diagnosis.

*P* <.05 is statistically significant.

ASA, American society of Anaesthesiologist; BMI, body mass index; GAD, generalised anxiety disorder; IQR, interquartile range, PHQ, patient health questionnaire .

**Table 2.** Number and Percentage of Patients Requiring Rescue Analgesia in Patients Receiving Pregabalin Versus Duloxetine

	Group Pregabalin n = 30	Group Duloxetine n = 30	*P*
No. of patients who used rescue analgesia n (%)	18 (60)	15 (50)	.44
No. of patients who used second rescue n (%)	2 (6. 6)	3 (10)	.14
Use of first rescue time (h)	8.00 [4.00-12.00 (4-24)]	8.00 [4.00-12.00 (4-24)]	.94

Values are represented as number and % for number and % of patients. Chi-square test is used for % of patients who used rescue analgesia. Values are represented as median [IQR (range)] for the use of first rescue analgesia. Mann–Whitney test is used for the calculation of use of first rescue analgesia.

*P* <.05 is statistically significant.

IQR, interquartile range.

## References

[1] Hussain G Wang J Rasul A et al. Current status of therapeutic approaches against peripheral nerve injuries: a detailed story from injury to recovery. Int J Biol Sci. 2020;16(1):116 134. 10.7150/ijbs.35653) 31892850 PMC6930373

[2] Borsook D Kussman BD George E Becerra LR Burke DW . Surgically-induced neuropathic pain (SNPP): understanding the perioperative process. Ann Surg. 2013;257(3):403 412. 10.1097/SLA.0b013e3182701a7b) 23059501 PMC3546123

[3] Wardhan R Chelly J . Recent advances in acute pain management: understanding the mechanisms of acute pain, the prescription of opioids, and the role of multimodal pain therapy. F1000Res. 2017;6:2065. 10.12688/f1000research.12286.1) PMC5710326 29225793

[4] Souzdalnitski D Narouze S . Clinical expertise in regional anesthesia: anesthesiologists voice their need for formal training. Saudi J Anaesth. 2013;7(4):371 372. 10.4103/1658-354X.121042) 24348285 PMC3858684

[5] Baidya DK Agarwal A Khanna P Arora MK . Pregabalin in acute and chronic pain. J Anaesthesiol Clin Pharmacol. 2011;27(3):307 314. 10.4103/0970-9185.83672) 21897498 PMC3161452

[6] Patetsos E Horjales-Araujo E . Treating chronic pain with SSRIs: what do we know? Pain Res Manag. 2016;2016:2020915. 10.1155/2016/2020915) PMC4947493 27445601

[7] Gaumann D Forster A Griessen M Habre W Poinsot O Della Santa D . Comparison between clonidine and epinephrine admixture to lidocaine in brachial plexus block. Anesth Analg. 1992;75(1):69 74. 10.1213/00000539-199207000-00013) 1616165

[8] Ladlow P Phillip R Coppack R et al. Influence of immediate and delayed lower-limb amputation compared with lower-limb salvage on functional and mental health outcomes post-rehabilitation in the U.K. Military. J Bone Joint Surg Am. 2016;98(23):1996 2005. 10.2106/JBJS.15.01210) 27926681

[9] Ramsay MA Acute postoperative pain management. Proc (Bayl Univ Med Cent). 2000;13(3):244 247. 10.1080/08998280.2000.11927683) 16389390 PMC1317048

[10] Ziemann-Gimmel P Goldfarb AA Koppman J Marema RT . Opioid-free total intravenous anaesthesia reduces postoperative nausea and vomiting in bariatric surgery beyond triple prophylaxis. Br J Anaesth. 2014;112(5):906 911. 10.1093/bja/aet551) 24554545

[11] Thambiah MD Nathan S Seow BZ Liang S Lingaraj K . Patient satisfaction after total knee arthroplasty: an Asian perspective. Singapore Med J. 2015;56(5):259 263. 10.11622/smedj.2015074) 26034317 PMC4447926

[12] Foss NB Kristensen MT Kehlet H . Prediction of postoperative morbidity, mortality and rehabilitation in hip fracture patients: the cumulated ambulation score. Clin Rehabil. 2006;20(8):701 708. 10.1191/0269215506cre987oa) 16944827

[13] Singh T Sharma S Nagesh S . Socio-economic status scales updated for 2017. Int J Res Med Sci. 2017;5(7):3264 3267. 10.18203/2320-6012.ijrms20173029)

[14] Chincholkar M Gabapentinoids: Pharmacokinetics, pharmacodynamics and considerations for clinical practice. Br J Pain. 2020;14(2):104 114. 10.1177/2049463720912496) 32537149 PMC7265598

[15] Bellingham GA Peng PW . Duloxetine: a review of its pharmacology and use in chronic pain management. Reg Anesth Pain Med. 2010;35(3):294 303. 10.1097/AAP.0b013e3181df2645) 20921842

[16] Altiparmak B Güzel Ç Gümüş Demirbilek S . Comparison of preoperative administration of pregabalin and duloxetine on cognitive functions and pain management after spinal surgery: a randomized, double-blind, placebo-controlled study. Clin J Pain. 2018;34(12):1114 1120. 10.1097/AJP.0000000000000640) 30020088

[17] Ferdinand RF Dieleman G Ormel J Verhulst FC . Homotypic versus heterotypic continuity of anxiety symptoms in young adolescents: evidence for distinctions between DSM-IV subtypes. J Abnorm Child Psychol. 2007;35(3):325 333. 10.1007/s10802-006-9093-0) 17226094 PMC1915634

[18] Li F Ma J Kuang M et al. The efficacy of pregabalin for the management of postoperative pain in primary total knee and hip arthroplasty: a meta-analysis. J Orthop Surg Res. 2017;12(1):49. 10.1186/s13018-017-0540-0) PMC5366132 28340617

[19] Wang YM Xia M Shan N et al. Pregabalin can decrease acute pain and postoperative nausea and vomiting in hysterectomy: a meta-analysis. Med (Baltim). 2017;96(31):e7714. 10.1097/MD.0000000000007714) PMC5626165 28767611

[20] Ho KY Tay W Yeo MC et al. Duloxetine reduces morphine requirements after knee replacement surgery. Br J Anaesth. 2010;105(3):371 376. 10.1093/bja/aeq158) 20573635

[21] Kassim DY Esmat IM Elgendy MA . Impact of duloxetine and dexamethasone for improving postoperative pain after laparoscopic gynecological surgeries: a randomized clinical trial. Saudi J Anaesth. 2018;12(1):95 102. 10.4103/sja.SJA_519_17) 29416464 PMC5789514

[22] Govil N Parag K Arora P Khandelwal H Singh A Ruchi . Perioperative duloxetine as part of a multimodal analgesia regime reduces postoperative pain in lumbar canal stenosis surgery: a randomized, triple blind, and placebo-controlled trial. Korean J Pain. 2020;33(1):40 47. 10.3344/kjp.2020.33.1.40) 31888316 PMC6944370

[23] Castro-Alves LJ Oliveira de Medeiros AC Neves SP et al. Perioperative duloxetine to improve postoperative recovery after abdominal hysterectomy: a prospective, randomized, double-blinded, placebo-controlled study. Anesth Analg. 2016;122(1):98 104. 10.1213/ANE.0000000000000971) 26421810

[24] Toth C Pregabalin: latest safety evidence and clinical implications for the management of neuropathic pain. Ther Adv Drug Saf. 2014;5(1):38 56. 10.1177/2042098613505614) 25083261 PMC4110876

[25] Li H Li T Li Y Shen Y . Pharmacokinetics and safety of duloxetine enteric-coated tablets in Chinese healthy volunteers: a randomized, open-label, single- and multiple-dose study. Clin Psychopharmacol Neurosci. 2013;11(1):28 33. 10.9758/cpn.2013.11.1.28) 23678352 PMC3650295

